# Bismuth-Based Metal–Organic Framework as a Chemiresistive Sensor for Acetone Gas Detection

**DOI:** 10.3390/nano13233041

**Published:** 2023-11-28

**Authors:** Ashraf Ali, Yaser E. Greish, Reem H. Alzard, Lamia A. Siddig, Ahmed Alzamly, Naser Qamhieh, Saleh T. Mahmoud

**Affiliations:** 1 Department of Physics, United Arab Emirates University, Al-Ain 15551, United Arab Emirates; 2 Department of Chemistry, United Arab Emirates University, Al-Ain 15551, United Arab Emirates

**Keywords:** acetone sensor, breath analyzer, Bi-gallate MOF, chitosan

## Abstract

Analyzing acetone in the exhaled breath as a biomarker has proved to be a non-invasive method to detect diabetes in humans with good accuracy. In this work, a Bi-gallate MOF doped into a chitosan (CS) matrix containing an ionic liquid (IL) was fabricated to detect acetone gas with a low detection limit of 10 ppm at an operating temperature of 60 °C and 5 V operating bias. The sensor recorded the highest response to acetone in comparison to other test gases, proving its high selectivity along with long-term stability and repeatability. The sensor also exhibited ultra-fast response and recovery times of 15 ± 0.25 s and 3 ± 0.1 s, respectively. Moreover, the sensor membrane also exhibited flexibility and ease of fabrication, making it ideal to be employed as a real-time breath analyzer.

## 1. Introduction

As the world advances in technology and industries, this has also caused pollution of the environment to accelerate. The polluted environment harbors diseases that affect the pace of human existence. Focusing on one form of pollution, in line with the advancement in technology, there has been an exponential decline in the quality of the air we breathe. Low quality of the air has adverse effects on our health and lifestyle. In response to this scenario, the diagnosis of diseases triggered by this poor air quality at early stages greatly increases the chances of early treatment and the betterment of the individual’s health.

Amazingly, the human breath incorporates a lot of gases that can divulge information on the health state, but only if we have a way to decipher the data. Hence, sensors that can detect and distinguish these gases in real-time offer an inexpensive, non-invasive approach to gaining diagnostic information on the diseases people may have developed from inhaling polluted air. The gases that are exhaled by humans mostly contain a mixture of CO_2_, N_2_, H_2_O, O_2_, and trace levels of other volatile organic compounds (VOCs) such as ethane or acetone, to name a few. These gases assist as biomarkers in the detection of many diseases such as diabetes, lung cancer, etc. [1,2,3,4,5]. Acetone is a product of a biological process between the human and the invading micro-organisms. It then infuses into the bloodstream and is transported to the lungs. From here, it becomes a key component in the exhaled breath [6], enabling us to detect its concentration, based on which we can non-invasively distinguish numerous diseases. According to the World Health Organization (WHO), the level of acetone in a healthy human is supposed to be 0.2–1.8 ppm, in contrast to a diabetic patient, in whom it is between 1.25 and 2.5 ppm [7]. Determining the concentrations in terms of parts per billion (ppb) has proved to be quite a challenge, but there have been a few materials [8,9] that have been up for the task.

Researchers have not only been making groundbreaking advances in materials with the potential for acetone detection, but they have also been using acetone as a cleaning agent and solvent for their experimentations [10,11]. However, it has been demonstrated that prolonged exposure to acetone vapors exceeding 173 ppm can cause long-term health issues such as central nervous system anesthesia, skin and eye irritation, narcosis, nausea, headaches, and dizziness [12,13,14,15,16]. It is also flammable and explosive, with lower and upper explosive limits of 2.6% and 12.8%, respectively [17].

Metal–organic frameworks (MOFs) have been demonstrated to be a key component in developing devices that can detect hazardous gases evolving due to various processes. MOFs consist of metal cations lined by organic ligand molecules [18,19]. With properties like high porosity and tunable surface area diversity in structures, they have various applications such as gas separation and storage [20], catalysis [21], and energy applications and as sensing materials [22,23,24,25]. However, there is one obstacle for some of them, which is related to their high electrical resistance. As standalone materials, they would not serve the purpose of their designs, but when they are combined into an organic–inorganic matrix with ionic liquids (ILs), they can exhibit changes in their properties that can be recorded and evaluated.

Sensors based on these materials tend to operate under various principles such as impedance sensing [26], chemicapacitive sensing [27], chemiresistive sensing, Kelvin probe [28], capacitive sensing [29], field effect transistors [30,31], optical sensing, fluorescence sensing for the detection of various materials such as volatile gases, hazardous gases, and explosive compounds [32,33,34], ion sensing [35], biosensing [36,37], humidity sensing [38,39,40], pH sensing [41,42], and temperature sensing [43]. There are rapid photophysical, electrical, or mechanical changes in the properties of the material, with various circumstances that influence the behavior depending on the concentration of the analyte, active materials, ability to bond, electron accepting–donating ability, and hydrogen bonding, to name a few [44]. From the aforementioned principles, one of the simplest is chemiresistive sensing, which measures the change in resistance of the sensing material when exposed to the target gas. The mechanism of sensing in these materials is attributed to the transfer of electrons or holes, which results in an interaction between the surface of the sensing material and the target gas molecules via adsorption or surface reactions. Other advantages that these chemiresistive sensors have to offer are low cost of fabrication, easy integration with other electronic components for commercial devices, low operational costs, and ease of miniaturization [23,45,46]. In addition, the ability to detect trace-level analytes with efficiency and accuracy enables them to be successfully commercialized.

The change in resistance in these sensing materials is dependent on the type of material. With the diversity in the MOF and MOF-based materials, the sensing mechanism can also be altered. The interaction of the test gas on the surface can either donate an electron to the material or deprive it of one, thereby causing a change in resistance [47]. The linkers or the active functional organic groups that are used in the synthesis of these materials serve as effective adsorption sites that facilitate the transfer of charges within the system. Depending on the test gas, the reduction or oxidization reaction [48], due to its interaction with the material, can also be the cause for the change in the resistance of the material. Another parameter is the change in volume of the MOF on interaction with the gas modulating the number of electrons that are transferred during the interaction [49]. Depending on the concentration of the gas, the response of the sensor is recorded. As the synthesis methods of the materials advance, the MOFs show enhanced conductivities and porosities that increase the sensitivities multifold.

Bismuth-based materials have been traditionally used for cosmetics and drug delivery. Wang et al. [50] reported an elaborate study deciphering the structure of the Bi-MOF. Furthermore, Z. Wang et al. [51] reported a detailed review of the Bi-based MOFs and their derivatives, outlining that they have been traditionally used in catalysis applications [21,52,53], electrocatalysis, sensors [54], CO_2_ capture [51], electrochemical energy storage [20,55], biomedical imaging [56], drug delivery, fluorescence sensing [51], absorption, and separation [52,57]. Mirica et al. [58], meanwhile, reported the synthesis and characterization of Bi-based MOFs for the detection of VOC compounds such as acetone, MeOH, and EtOH. They demonstrated that the material can also detect NO and NH_3_ at room temperature. Some of the reported sensors from the literature have been consolidated in Table 1 for comparison with our work.

Our group has demonstrated that chitosan (CS) polymer incorporation with glycerol, as an IL [65], can detect H_2_S gas at 15 ppm operating at 80 °C [24]. Conventionally, it has also been used in combination with MOFs and ZIFs [25,59] to enhance the detection of H_2_S at room temperature. To the best of the authors’ knowledge, the Bi-gallate MOF has not been used as an acetone sensor in combination with an organic matrix. Hence, in this work, we present the possibility of employing the Bi-gallate MOF in combination with CS/IL matrix as an acetone sensor with a breath analyzer application for the detection of diabetic patients.

## 2. Materials and Methods

### 2.1. Materials

Bismuth (III) nitrate pentahydrate (Bi(NO_3_)_3_·5H_2_O), gallic acid (3,4,5-trihydroxybenzoic acid), and anhydrous dimethylformamide (DMF) were bought from Sigma-Aldrich, St. Louis, MO, USA. Chitosan (MW = 50,000–190,000 Da) (≥75%) and acetic acid were purchased from Polysciences, Warrington, PA, USA. Glycerol, as an ionic liquid (IL) (99.5%), was purchased from Quarek Corp company, Denver, CO, USA. All chemicals were used without further purification.

### 2.2. Synthesis of Bi-Gallate MOF

Bi-gallate MOF was synthesized following a slightly modified version of a previously published procedure [50]. In a scintillation vial, gallic acid (17 mg, 2 mmol) was dissolved in 3 mL of deionized water. Then, drops of 4 M NH_4_OH were added to the solution until the pH reached 8.5. In a separate scintillation vial, Bi(NO_3_)_3_·5H_2_O (24 mg, 1 mmol) was dissolved in 1 mL of DMF and then gradually added with continuous stirring to the gallic acid solution. The vial was sealed and placed in a preheated oven set at 85 °C for a duration of 24 h. After completion of the reaction, the resulting yellow powder was filtered and successively washed with distilled water and ethanol. Then, the Bi-gallate compound was subjected to activation in a vacuum oven at 90 °C for 24 h to remove any trapped solvent molecules.

### 2.3. Fabrication of the Bi-Gallate MOF/CS/IL Membrane

**Figure 1:**
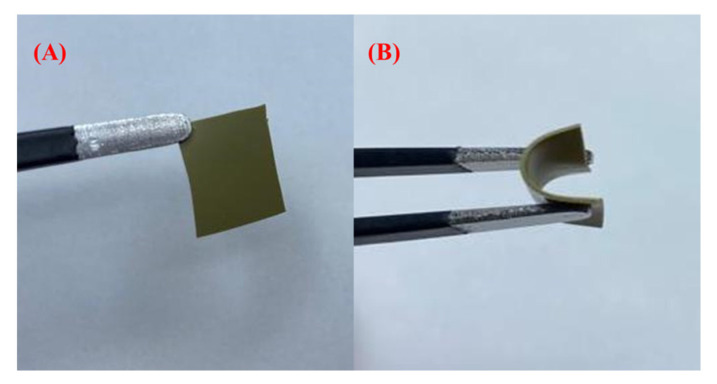
(**A**) A 1 cm × 1 cm portion of the membrane. (**B**) Demonstration of high flexibility.

The Bi-gallate MOF/CS/IL membrane was fabricated by dissolving 2 wt% (0.4 g) of the Bi-gallate MOF with 0.4 g of CS and 5 vol% of IL into 20 mL of 3% acetic acid solution. The solution was then stirred for 24 h at room temperature and 1400 RPM on a magnetic stirrer. The prepared solution was then cast on a Petri dish and dried at 70 °C for 18 h in a drying oven. A flexible and uniform membrane formed, as shown in Figure 1. The thickness of the membrane was determined to be 0.20 mm using a screw gauge.

### 2.4. Characterization

Powder X-ray diffraction (PXRD) was conducted using a Rigaku MiniFlex X-ray diffractometer (Rigaku, Tokyo, Japan) with a CuKα radiation tube (wavelength = 1.542 Å) operated at 40 kV. PXRD measurements were taken within a 3° to 50° 2θ range at a rate of 2° min**^−^**^1^. Scanning electron microscopy (SEM) was performed using JSM-6010LA, JEOL, Tokyo, Japan, with an operating voltage of 5 keV. The secondary electron imaging configuration was used to procure the images with a working distance of 14 mm. The samples were made conductive with a gold sputtering system. The surface area and porosity were examined using nitrogen sorption analysis at 77 K. The N_2_ adsorption–desorption isotherm indicated gas adsorption (cm^3^ g**^−^**^1^) in relation to relative pressure (P/P_0_). Here, P represents N_2_ equilibrium pressure, and P_0_ represents saturated vapor pressure at 77 K. Prior to surface measurements, powder samples were placed in a glass tube and subjected to vacuum at 353 K for 3 h. Thermogravimetric analysis (TGA) was performed using a TGA-50 Shimadzu analyzer (Shimadzu, Kyoto, Japan) with an aluminum pan sample holder. FTIR analysis was carried out using a Shimadzu IRAffinity-1S with a scan range from 400 to 4000 cm**^−^**^1^.

### 2.5. Sensor Fabrication and Gas Testing

The sensor prototype was fabricated by sandwiching the active layer between a Cu plate with dimensions 1.5 cm × 1.5 cm as the bottom electrode and a stainless steel mesh with a grid size of 250 μm × 250 μm serving as the top electrode [59,66]. The layers were confined using temperature-resistive Kapton tape. The device was connected to the sensing system with electrical probes which were housed in a Teflon chamber connected to Bronkhorst mass flow controllers (MFCs). The setup was sealed to avoid leaking of the test gas and placed inside the fume hood for safety. The humidity of the chamber was maintained close to 0% throughout the testing sequences. The gas testing programs were sequenced to expose the sample to test gas in between cycles of synthetic air to exfoliate any residual test gas molecules.

## 3. Results and Discussion

### 3.1. Structural and Morphological Characterization of Bi-Gallate MOF and Bi-MOF/CS/IL Membrane

**Figure 2:**
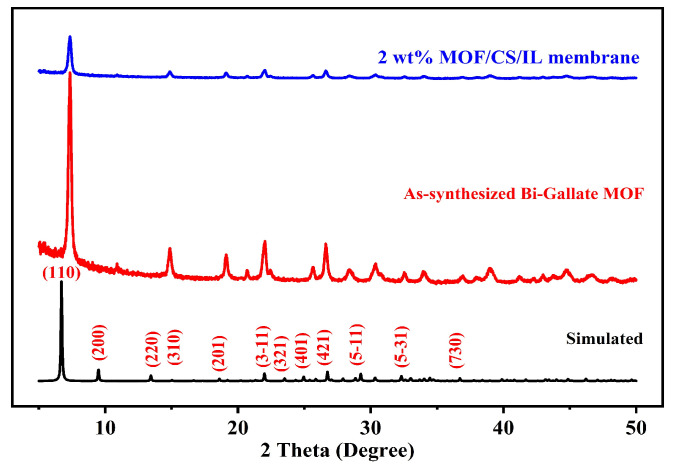
PXRD pattern of the Bi-gallate MOF and the composite membrane.

The structural analysis of the Bi-gallate MOF and Bi-gallate MOF/CS/IL membranes was carried out using the XRD patterns obtained. The XRD pattern in Figure 2 confirmed the structure and phase purity of the synthesized Bi-gallate MOF. By comparing the resulting diffraction pattern with the simulated pattern derived from single-crystal data as previously reported [50], it was confirmed that the Bi-gallate we prepared was successfully and purely synthesized. The Bi-gallate material exhibited an orthorhombic lattice and unit cell dimensions of a = 8.80 Å, b = 4.66 Å, c = 24.09 Å. The XRD pattern of the Bi-gallate MOF/CS/IL with a broad hump confirmed the incorporation of the Bi-gallate MOF into the CS matrix, as can be seen in the top pattern in Figure 2. The characteristic peaks representing the (110), (310), (321), and (421) planes were observed.

**Figure 3:**
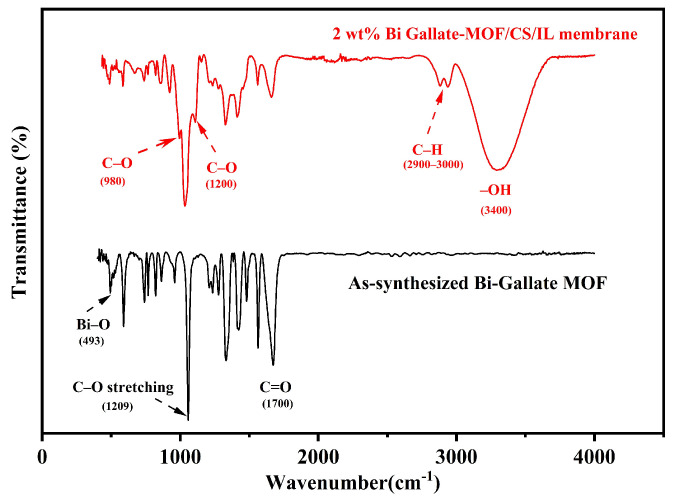
FTIR curves of the as-synthesized Bi-gallate MOF powder and the composite membrane.

The FTIR analysis of Bi-gallate MOF and Bi-gallate MOF/CS/IL membrane confirmed the interaction within the framework of Bi-gallate and confirmed its presence within the composite, as shown in Figure 3. The disappearance of the hydroxyl group (-OH) band confirmed the chelation of bismuth metal with the gallic acid linker. Furthermore, a band was observed around 1671 cm**^−^**^1^, which is attributed to the presence of (C=O) of a carboxylate (COO^−^) group. Analyzing the spectra of the Bi-gallate MOF/CS/IL membrane, a broad band at 3400 cm**^−^**^1^ was observed in the FTIR spectrum, and is attributed to the NH and OH stretching vibration, as well as the intermolecular hydrogen bonds of chitosan. Additionally, the bands at 2900 and 3000 cm**^−^**^1^ belong to the symmetric and asymmetric stretching of the C-H bond of chitosan, respectively. Meanwhile, the C-O bending and C-O stretching vibrations of chitosan were determined at 1100 and 980 cm**^−^**^1^, respectively. The presence of Bi-gallate MOF within the chitosan membrane was further confirmed through the sharp bands at 1700 and 1209 cm**^−^**^1^, which, respectively, belong to C=O and C-O stretching of the gallate linker. As can be observed in Figure 3, the band frequencies that are attributed to the carboxylate group (at 1671 cm**^−^**^1^) and the Bi-O group (at 439 cm**^−^**^1^) remained intact, which also confirms the stability of the MOF framework within the chitosan membrane.

**Figure 4:**
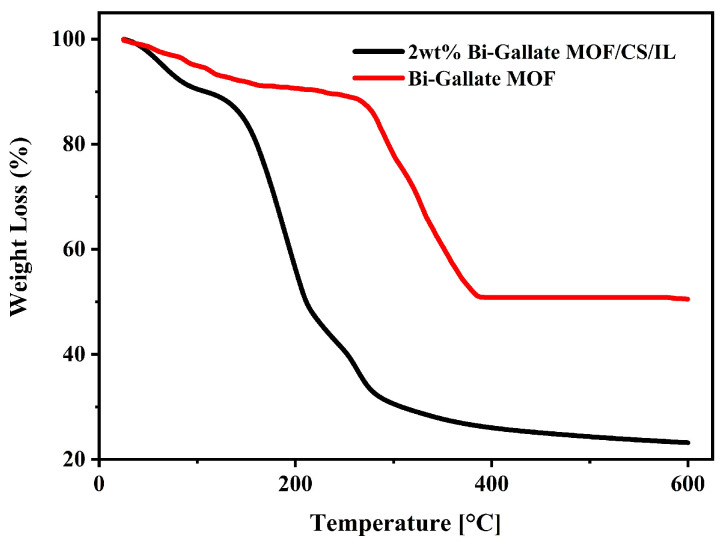
TGA curves of the Bi-gallate MOF powder and the composite membrane.

The thermal analysis of the as-synthesized Bi-gallate MOF was performed using TGA under a nitrogen atmosphere (Figure 4). In the temperature range of room temperature to 150 °C, the sample experienced a weight reduction of approximately 10%, corresponding to the loss of water molecules. This finding aligned with previously reported results [50]. Decomposition of the Bi-gallate commenced in the range of 260–350 °C, and is attributed to the breakdown of the gallate linker of the MOF structure. From the TGA curves of the Bi-gallate MOF/CS/IL membrane, we can surmise that the first weight loss observed would be due to the loss of water molecules from the pores of the framework. The drastic loss in weight observed between 150 °C and 225 °C indicates the onset of the decomposition of the gallate linker from the matrix, followed by a gradual loss in weight due to the continued decomposition of the remaining linkers.

**Figure 5:**
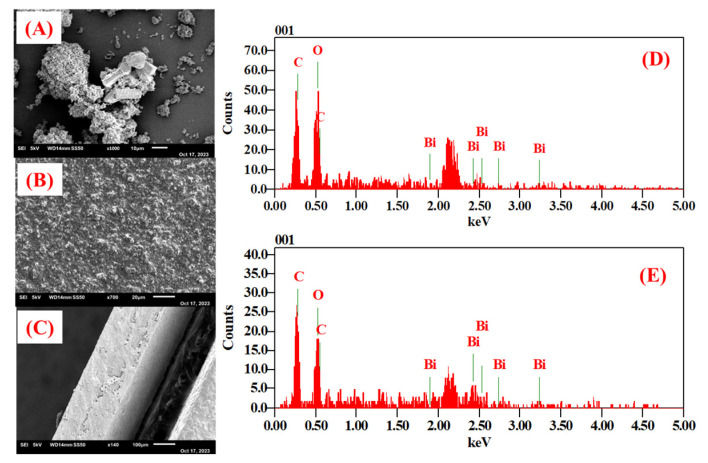
(**A**,**D**) SEM image and EDX spectra of the as-synthesized Bi-gallate MOF. (**B**,**E**) SEM image and EDX spectra of the Bi-gallate MOF/CS/IL membrane. (**C**) Cross-section of the membrane.

The SEM analysis of the Bi-gallate MOF powder, shown in Figure 5A, shows that the morphology of the as-synthesized powder is comparable to the reported morphologies of the same MOF [50]. The SEM micrographs of the Bi-gallate MOF/CS/IL membranes shown in Figure 5B displayed the incorporation of the MOFs into the matrix. The cross-section of the membrane showed numerous pores, as can be seen in Figure 5C. The EDX spectra we recorded of the as-synthesized MOF (Figure 5D) and the composite membrane (Figure 5E) showed the homogeneous distribution of the Bi-gallate MOF particles within the chitosan matrix.

**Figure 6:**
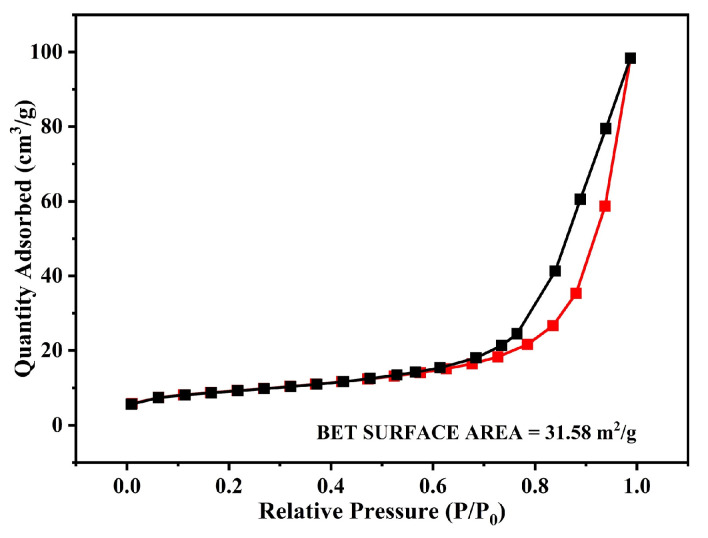
N_2_-sorption isotherms of as-synthesized Bi-gallate MOF.

The as-synthesized Bi-gallate MOF powder was also subjected to N_2_ adsorption measurements to evaluate the micro- and macroporosity in the MOF. The recorded loop shown in Figure 6 follows a type I isotherm with a calculated Brunauer–Emmett–Teller (BET) surface area of 31.58 m**^2^**/g, and shows a maximum pore volume of 0.0135 cm**^3^**/g.

### 3.2. Gas Sensing Performance

The sensor prototype was set up as detailed in our previous reports [59,66]. The CS/IL matrix was doped with different concentrations of Bi-gallate MOF. The membranes were subjected to different test gases at 100 ppm to evaluate their response. It was evaluated that 2 wt% doping of the Bi-gallate MOF into the CS/IL matrix was most sensitive toward acetone gas at an operating temperature of 60 °C and a bias voltage of 5 V. The response of the sensor was evaluated using Equation (1):

(1) $$S\;\left(\%\right)=\frac{R_{g}-R_{a}}{R_{a}}\times 100=\frac{∆R}{R_{a}}\times 100$$

where *R_a_* is the resistance of the sensor in synthetic air and *R_g_* is the resistance in the presence of the test gas.

**Figure 7:**
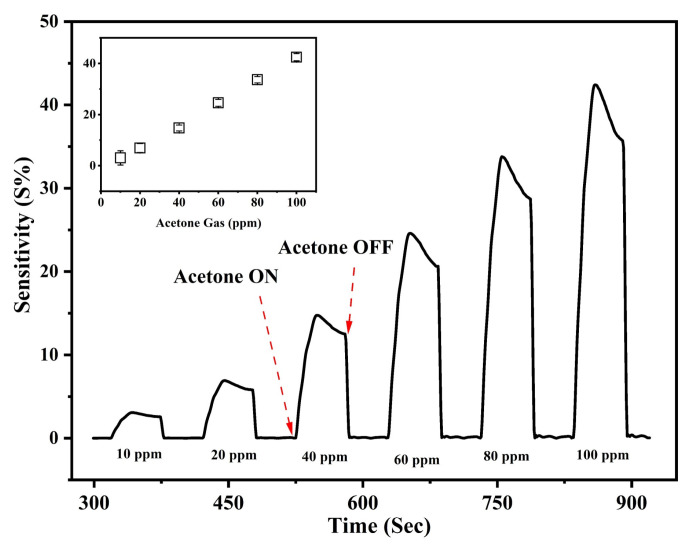
Sensitivity of the Bi-gallate MOF/CS/IL membrane as a function of time and acetone concentration measured at 60 °C. Inset: Sensitivity for corresponding gas concentration.

The sensor was evaluated in terms of sensitivity, which showed a response to 10 ppm of acetone gas at 60 °C, as plotted in Figure 7. The inset graph shows the sensor’s response toward different concentrations of acetone gas. The sensor was further analyzed toward other test gases at 100 ppm and an operating temperature of 60 °C.

**Figure 8:**
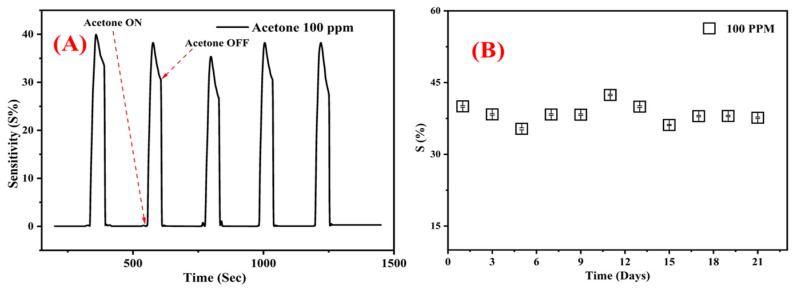
(**A**) Repeatability of the sensor. (**B**) Long-term stability of the Bi-gallate MOF/CS/IL membrane to 100 ppm of acetone gas at 60 °C.

The other aspects of evaluation were repeatability and stability. The tests were performed with exposure to 100 ppm of acetone, with synthetic air flushing in between each cycle to exfoliate any residual molecules from the previous cycle. The stability response was calculated as 42.41 ± 1.8%, whereas the repeatability of the sensor was calculated as 39.95 ± 1.4% (Figure 8).

**Figure 9:**
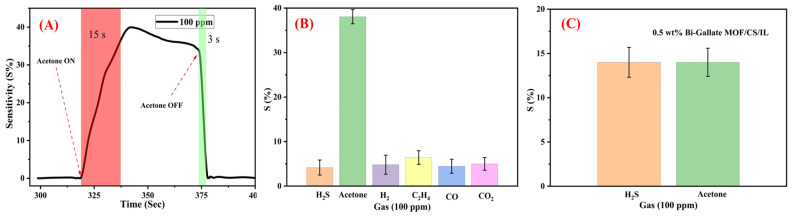
(**A**) The calculated response and recovery time of the sensor. (**B**) Selectivity of the sensor containing 2 wt% Bi-gallate MOF in comparison to other test gases. (**C**) Selectivity of the sensor containing 0.5 wt% Bi-gallate MOF to H_2_S and acetone gases.

Another aspect of evaluating the sensor’s performance is the response and recovery times, which can be defined as the time taken from gas exposure for the sensor to reach 90% of its recorded response, and the time taken for the sensor to recover to 10% of its initial resistance from the shutdown of the gas, respectively. From the results shown in Figure 9A, the responses recorded were calculated as 15 ± 0.25 s and 3 ± 0.1 s, respectively. Yet another vital parameter is the selectivity among other test gases. It was recorded that the sensitivity toward acetone was the highest among other test gases such as H_2_, H_2_S, C_2_H_4_, CO, and CO_2_, as shown in Figure 9B.

### 3.3. Gas Sensing Mechanism

**Figure 10:**
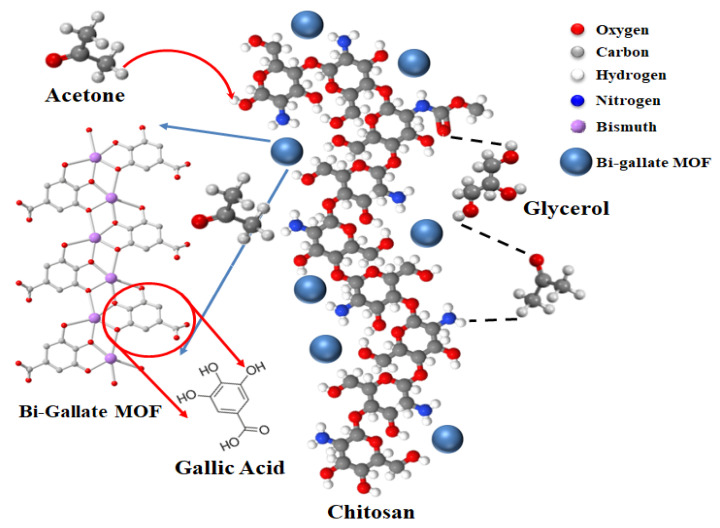
Sensing mechanism of the Bi-gallate-doped CS-IL membrane.

The mechanism of the standalone CS–IL membrane was outlined by Hani et al. [24]. The functional groups along the chitosan polymeric matrix (-OH and -NH_2_) interact with the highly hydroxylated IL molecules through the formation of a network of H bonding. In the presence of hydroxy- and carboxy-functionalized Bi-gallate MOF particles in the matrix, an extended network of H bonding is produced, as shown in Figure 10. Compared with slightly polar or non-polar gas molecules, H_2_S and acetone showed adsorption to this H-bonded network by taking part in the network through H bonding, as well. This was observed when a low proportion of the Bi-gallate MOF (0.5 wt%) was added, as shown in Figure 9C. Upon increasing the proportion of Bi-gallate MOF to 2 wt%, a higher tendency of acetone to adsorb onto the extensively formed H-bonded network was observed, as shown in Figure 9B. Compared with H_2_S molecules with a dipole moment of 0.95 D, the preferential adsorption of acetone molecules is attributed to its higher polarity (with a dipole moment of up to 4.19 D). It should be mentioned that the highly polar acetone molecules interact with the H-bonded network of chitosan containing IL and Bi-gallate through the attraction of its highly polar C=O group to the H atoms along the other components of the sensor. Accordingly, an increase in the sensitivity of the composite sensor membrane was observed when increasing the Bi-gallate component of the sensor.

## 4. Conclusions

This study demonstrates the potential of fabricating a fast response and ultra-fast recovery sensor based on Bi-gallate MOF doped into a chitosan matrix containing IL. The fabricated membrane was investigated for its sensing performance. Previous studies conducted by our group demonstrated that CS/IL membranes showed potential for sensing toward H_2_S gas, and doping the matrix with 2 wt% Bi-gallate MOF showed sensitivity toward acetone vapor alone with a 5 V bias. The detection limit of the prototype is 10 ppm of acetone at 60 °C, with ultra-fast response and recovery times of 15 ± 0.25 s and 3 ± 0.1 s, respectively. The components of the membrane do not cause any harm to the environment, hence making the prototype highly eco-friendly. The proposed membrane can be used as an acetone sensor with ultra-fast response and recovery times, which can be used as a real-time breath analyzer.

## Figures and Tables

**Table 1:** Sensor performance comparison with literature-reported values.

Sensor/Material	Target Gas	Optimum Operating Temperature (°C)	Detection Limit (ppm)	Ref.
Bi-gallate MOF/CS/IL membrane	Acetone	60	10	This Work
MOF-5/CS/IL membrane	H_2_S	RT	1	[59]
ZIF-67	Acetone	220	100	[60]
		250	50	[61]
ZIF-67/ZIF-8	Acetone	275	1	[62]
ZnO/ZIF-CoZn	Acetone	250	10	[63]
Hierarchical MOF derived ZnO-Co_3_O_4_	Acetone	450	5	[64]
Bi(HHTP)	NH_3_	RT	0.29	[58]
	NO	RT	0.15	
	Acetone	RT	41.2	
	MeOH	RT	278	
	EtOH	RT	185	

## Data Availability

The data that support the findings of this study are available from the corresponding authors upon request.
